# P-279. An Online Registry of Carbapenemase Producing Gram Negative Organisms for Evaluating the Clinical Profile, Outcomes and Mechanisms of Resistance in Two Tertiary Level Hospitals (ORiGiN Study): An Interim Analysis

**DOI:** 10.1093/ofid/ofae631.482

**Published:** 2025-01-29

**Authors:** Adrian Ronald A Espino, Danice Faith B Alombro, Justin Allister G Ong, Christel M Valdez, Shanaia Esthelle Joy Daguit, Daniel Jose N Ombao, Lawrence S Macalalad, Mark Carascal, Marc Agnew Cajucom, Anna Flor Malundo, Cybele Lara R Abad

**Affiliations:** University of the Philippines- Philippine General Hospital, Paranaque, National Capital Region, Philippines; University of the Philippines- Philippine General Hospital, Paranaque, National Capital Region, Philippines; The Medical City, Pasig, National Capital Region, Philippines; Consultant, Pasig, National Capital Region, Philippines; Clinical and Translational Research Institute, The Medical City, Quezon City, National Capital Region, Philippines; Biomedical Research Unit - The Medical City, Manila, National Capital Region, Philippines; The Medical City, Pasig, National Capital Region, Philippines; Biomedical Research Unit- Clinical and Translational Research Institute- The Medical City, Manila, National Capital Region, Philippines; University of the Philippines- Philippine General Hospital, Paranaque, National Capital Region, Philippines; University of the Philippines- Philippine General Hospiral, Manila, National Capital Region, Philippines; University of the Philippines - Philippine General Hospital, Manila, National Capital Region, Philippines

## Abstract

**Background:**

Infections with gram-negative carbapenem-resistant organisms (CRO) are an emerging global problem, but little is known about CRO in the Philippines. This study aims to build a registry and describe the clinical profile and molecular mechanisms of resistance of carbapenem-resistant infections.

**Figure:**
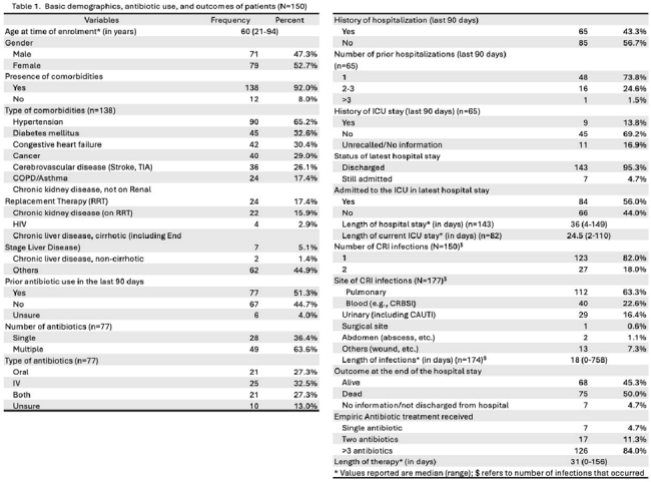

**Methods:**

This prospective study will enroll patients from two tertiary-level hospitals from 01/01/2023 to 12/31/2025. Admitted patients >18 years old with documented CRO infection confirmed by phenotypic resistance and molecular testing are included in this year-1 analysis.

**Figure:**
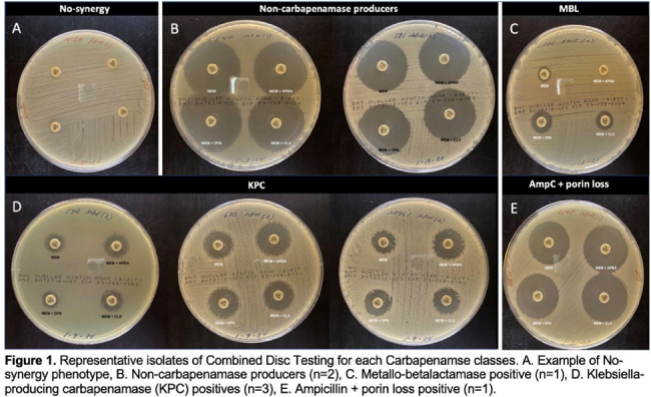

**Results:**

150 patients were enrolled in the first year. Around half (79/150, 54.7%) were female. Median age was 60 (range 21-94) years. Many had at least one comorbidity -- hypertension (90/150 65.2%), diabetes mellitus (45/150, 32.6%), and congestive heart failure (42/150, 30.4%). Most patients experienced CR infection only once (123/150, 82%), while a few (27/150,18%) had two or more. Infections originated from respiratory (63.3%), blood (22.6%), or urinary (16.4%) sites. Higher mortality was seen among patients who received three or more antibiotics (119/150, 57.1%) or had prolonged treatment ( >15 days) during admission (53.1%). Resistance to fluoroquinolones (OR_adjusted_=4.32 (95% CI: 1.11, 16.81); p=0.035) and older tetracyclines (OR_adjusted_=5.49 (95% CI: 1.08, 27.93); p=0.040) were significantly associated with mortality in the adjusted models. Only 9 out of 169 (5.32%) were Extended Spectrum Beta Lactamase (ESBL) producing. 40/169 (23.7%) were tested for carbapenem resistance genes and the two most common antimicrobial resistance genes were *bla_ndm_* (n= 17) and *bla_oxa51_* (n=20).

**Figure:**
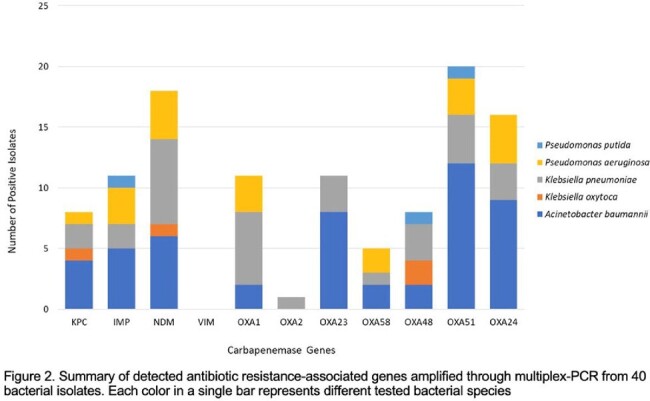

**Conclusion:**

The study provides initial data on the local epidemiology and molecular mechanisms of resistance of gram-negative CRO. Interim results show that CR infections affect the older population and are mainly respiratory in origin. Multiple antibiotics, prolonged therapy, and the use of fluoroquinolones and tetracyclines were all associated with higher mortality. Class D (OXA-51) and Class B (NDM) beta-lactamases were most frequently isolated.

**Disclosures:**

**All Authors**: No reported disclosures

